# Supplementary material for: Unveiling community patterns and trophic niches of tropical and temperate ants using an integrative framework of field data, stable isotopes and fatty acids
Source: PeerJ. 2018 Aug 22;6:e5467. doi: 10.7717/peerj.5467 (PMC6109374; doi:10.7717/peerj.5467)
Supplement: Supplemental Information 1 [file peerj-06-5467-s001.pdf]

Unveiling community patterns and trophic niches of tropical and temperate ants using an integrative framework of field data, stable isotopes and fatty acids

Felix Baumgarten Rosumek[1,2], Nico Blüthgen[1], Adrian Brückner[1,3], Florian Menzel[4], Gerhard Gebauer[5], Michael Heethoff[1]

Supplementary document S1: Bait composition and display (based on Houadria et al. 2015 and Rosumek 2017)

(1) Large prey: one individual of *Achaeta domesticus* (Linnaeus, 1758) with 1-2 cm body size, was tied between the first and second pair of legs to prevent it from escaping, but still be able to jump and move inside the trap. Represents larger, mobile prey with harder integument.

(2) Small prey: in Brazil, 15 termites were left free on a piece of mound, divided between workers and soldiers. Previous testing showed that termites do not leave the mound piece after 90 minutes. Another 10 individuals were glued to a small wood stick, to increase the time spent by ants on the bait. Individuals from the subfamily Nasutitermitinae were used, which have specialized workers with chemical defenses that can affect ants (Prestwich, 1984). In Germany, termites are rare, thus we used larvae of *Lucilia sericata* Meigen, 1826. Two maggots were pinned at the rear end, still able to move the body but not leave the bait. Previous testing showed

that they stayed alive for several hours under this condition. Both baits represent prey smaller, slower and softer, but not defenseless.

(3) Dead arthropods (2-3 g): crushed crickets and mealworms (*Tenebrio molitor* Linnaeus, 1758 – in Brazil) or maggots (*Lucilia sericata* Meigen, 1826 – in Germany).

(4) Bird feces (2-3 g): chicken feces from organic breeding. Represents nitrogen-rich bird feces, which could be directly collected by ants, or contain animal and vegetal remains used by them (Leal & Oliveira, 1998; Jaffe et al., 2001; Sainz-Borgo, 2015).

(5) Seeds (2-3 g): in Brazil, a mix of soy, millet, sunflower, canary, barley, linseed, grass seeds and ground corn, representing different sizes and shapes of seeds. We avoided seeds with elaiosomes because these mimic animal prey and attract predacious species, not only granivorous ones (Hughes et al., 1994; Giladi, 2006). However, in Germany, granivory is restricted to *Messor* ants, absent in our study site (Seifert, 2007). Thus, we used elaiosome seeds of *Chelidonium majus* (L.), known to be attractive to ants (Reifenrath, Becker & Poethke, 2012).

(6) Sucrose solution (2-3 ml): at 20% concentration. Sucrose and its components, fructose and glucose, are the main nutritional sugars in plant exudates and fleshy fruits (Percival, 1961; Baker et al., 1998; Blüthgen et al., 2004).

(7) Melezitose solution (2-3 ml): at 20% concentration. Represents insect-synthesized oligosaccharides present on honeydew (Kiss, 1981; Wäckers, 2000). Although melezitose and other oligosaccharides occur naturally almost only on honeydew, some insects secrete honeydew with little modification, similar to plant exudates (Völkl et al., 1999; Blüthgen et al., 2004). Therefore, preference for melezitose indicates use of honeydew, and avoidance suggests limited use, although not definitive lack of interaction with sap-sucking insects.

References

Blüthgen N, Gottsberger, G, Fiedler K. 2004. Sugar and amino acid composition of ant-attended nectar and honeydew sources from an Australian rainforest. *Austral Ecology* 29:418–429 DOI 10.1111/j.1442-9993.2004.01380.x.

Giladi I. 2006. Choosing benefits or partners: a review of the evidence for the evolution of myrmecochory. *Oikos* 112:481–492 DOI 10.1111/j.0030-1299.2006.14258.x.

Houadria M, Salas-Lopez A, Orivel J, Blüthgen N, Menzel F. 2015. Dietary and temporal niche differentiation in tropical ants—can they explain local ant coexistence? *Biotropica* 47:208–217 DOI 10.1111/btp.12184.

Hughes L, Westoby M, Jurado E. 1994. Convergence of elaiosomes and insect prey: evidence from ant foraging behaviour and fatty acid composition. *Functional Ecology* 8:358–365.

Jaffe K, Caetano FH, Sánchez P, Hernández JV, Caraballo L, Vitelli-Flores J, Monsalve W, Dorta B, Lemoine VR. 2001. Sensitivity of ant (*Cephalotes*) colonies and individuals to antibiotics implies feeding symbiosis with gut microorganisms. *Canadian Journal of Zoology* 79:1120–1124 DOI 10.1139/z01-079.

Kiss A. 1981. Melezitose, aphids and ants. *Oikos* 37:382–382.

Leal IR, Oliveira PS. 1998. Interactions between fungus-growing ants (Attini), fruits and seeds in Cerrado vegetation in Southeast Brazil. *Biotropica* 30:170–178 DOI 10.1111/j.1744-7429.1998.tb00052.x.

Prestwich GD. 1984. Defense mechanisms of termites. *Annual Review of Entomology* 29:201–232.

Reifenrath K, Becker C, Poethke HJ. 2012. Diaspore trait preferences of dispersing ants. Journal of Chemical Ecology 38:1093–1104 DOI 10.1007/s10886-012-0174-y.

Rosumek FB. 2017. Natural history of ants: what we (do not) know about trophic and temporal niches of neotropical species. *Sociobiology* 66:244–255 DOI 10.13102/sociobiology.v64i3.1623.

Sainz-Borgo C. 2015. Bird feces consumption by fire ant *Solenopsis geminata* (Hymenoptera: Formicidae). *Entomological News* 124:295–299 DOI 10.3157/021.124.0408.

Seifert B. 2007. *Die Ameisen Mittel- und Nordeuropas*. Tauer: Lutra.

Völkl W, Woodring J, Fischer M, Lorenz MW, Hoffmann KH. 1999. Ant-aphid mutualisms: the impact of honeydew production and honeydew sugar composition on ant preferences. *Oecologia* 118:483–491 DOI 10.1007/s004420050751.

Wäckers FL. 2000. Do oligosaccharides reduce the suitability of honeydew for predators and parasitoids? A further facet to the function of insect-synthesized honeydew sugars. *Oikos* 90:197–201 DOI 10.1034/j.1600-0706.2000.900124.x.
