# Supplementary material for: Unveiling community patterns and trophic niches of tropical and temperate ants using an integrative framework of field data, stable isotopes and fatty acids
Source: PeerJ. 2018 Aug 22;6:e5467. doi: 10.7717/peerj.5467 (PMC6109374; doi:10.7717/peerj.5467)
Supplement: Supplemental Information 3 [file peerj-06-5467-s003.pdf]

Incidence of species recorded in this work and labels used in plots.

Species	IB	IP	Label	Species	IB	IP	Labels
**Brazil**							
Acanthognathus brevicornis Smith, 1944	-	-	acanbr	*Pheidole schmalzi* Emery, 1894	-	1	pheisc
Acanthognathus ocellatus Mayr, 1887	-	-	acanoc	*Pheidole sigillata* Wilson, 2003	33	18	pheisi
Acromyrmex aspersus (Smith, 1858)	1		acroas	*Pheidole* sp.1	18	13	phei01
Acromyrmex laticeps (Emery, 1905)	-	-	acrola	*Pheidole* sp.2	57	38	phei02
Acromyrmex lundii (Guérin-Méneville, 1838)	-	-	acrolu	*Pheidole* sp.4	17	23	phei04
Acromyrmex subterraneus (Forel, 1893)	-	-	acrosu	*Pheidole* sp.5	1	2	phei05
Apterostygma acre Lattke 1997	1	-	apteac	*Pheidole* sp.6	4	2	phei06
Azteca sp.1	-	1	azte01	*Pheidole* sp.7	5	6	phei07
Azteca sp.2	-	-	azte02	*Pheidole* sp.8	-	1	phei08
Brachymyrmex sp.1	2	1	brac01	*Pheidole* sp.9	-	1	phei09
Brachymyrmex sp.2	-	1	brac02	*Pheidole* sp.10	1	-	phei10
Camponotus lespesii Forel, 1886	4	1	cample	*Pseudomyrmex flavidulus* (Smith, 1858)	-	1	pseufl
Camponotus zenon Forel, 1912	10	0	campze	*Solenopsis subterranea* MacKay & Vinson, 1989	-	1	solesu
Cephalotes pallidicephalus (Smith, 1876)	-	-	cephpa	*Solenopsis* sp.1	41	20	sole01
Cephalotes pusillus (Klug, 1824)	-	-	cephpu	*Solenopsis* sp.2	43	28	sole02
Crematogaster nigropilosa Mayr, 1870	4	1	cremni	*Solenopsis* sp.3	12	8	sole03
Cyphomyrmex rimosus (Spinola, 1851)	6	10	cyphri	*Solenopsis* sp.4	40	24	sole04
Gnamptogenys striatula Mayr, 1884	21	21	gnamst	*Solenopsis* sp.5	1	1	sole05
Fulakora elongata (Santschi, 1912)	1	-	fulael	*Solenopsis* sp.6	12	7	sole06
Heteroponera dentinodis (Mayr, 1887)	1	1	hetede	*Solenopsis* sp.7	-	1	sole07
Heteroponera dolo (Roger, 1860)	-	1	hetedo	*Solenopsis* sp.8	7	2	sole08
Hypoponera sp.1	-	2	hypo01	*Strumigenys cosmostela* Kempf, 1975	-	1	struco
Hypoponera sp.2	-	1	hypo02	*Strumigenys denticulata* Mayr, 1887	-	6	strude
Hypoponera sp.3	-	1	hypo03	*Strumigenys elongata* Roger, 1863	-	1	struel
Hypoponera sp.4	1	-	hypo04	*Strumigenys splendens* (Borgmeyer, 1954)	-	1	strusp
Hylomyrma reitteri (Mayr, 1887)	7	9	hylore	*Tapinoma atriceps* Emery, 1888	-	1	tapiat
Linepithema iniquum (Mayr, 1870)	7	-	linein	*Trachymyrmex* sp.1	1	1	trac01
Linepithema leucomelas (Emery, 1894)	-	2	linele	*Wasmannia affinis* Santschi, 1929	6	2	wasmaf
Linepithema micans (Forel, 1908)	6	1	linemi	*Wasmannia auropunctata* (Roger, 1863)	16	0	wasmau
Linepithema pulex Wild, 2007	4	1	linepu	*Wasmannia lutzi* Forel, 1908	-	1	wasmlu
Neoponera crenata (Roger, 1861)	-	1	neopcr	**Germany**			
Neoponera villosa (Fabricius, 1804)	-	1	neopvi	*Formica cunicularia* (Latreille, 1798)	2	5	formcu
Nylanderia docilis (Forel, 1908)	1	-	nylado	*Formica fusca* Linnaeus, 1758	35	41	formfu
Nylanderia sp.1	54	23	nyla01	*Formica rufibarbis* Fabricius, 1793	-	1	formru
Octostruma petiolata (Mayr, 1887)	2	2	octope	*Lasius brunneus* (Latreille, 1798)	4	2	lasibr
Octostruma stenognatha Brown & Kempf, 1960	-	1	octost	*Lasius flavus* (Fabricius, 1782)	-	12	lasifl
Octostruma sp.1	1	1	octo01	*Lasius fuliginosus* (Latreille, 1798)	5	5	lasifu
Odontomachus chelifer (Latreille, 1802)	20	11	odonch	*Lasius niger* (Linnaeus, 1758)	25	26	lasini
Odontomachus meinerti Forel, 1905	1	1	odonme	*Lasius platythorax* Seifert, 1991	10	10	lasipl
Oxyepoecus plaumanni Kempf, 1974	-	1	oxyepl	*Myrmica lobicornis* Nylander, 1846	1	5	myrmlo
Pachycondyla harpax (Fabricius, 1804)	1	10	pachha	*Myrmica rubra* (Linnaeus, 1758)	15	22	myrmrb
Pachycondyla striata Smith, 1858	43	40	pachst	*Myrmica ruginodis* Nylander, 1846	25	16	myrmrg
Pheidole angusta Forel, 1908	3	1	pheian	*Myrmica rugulosa* Nylander, 1849	1	2	myrmrl
Pheidole aper Forel, 1912	9	7	pheiap	*Myrmica scabrinodis* Nylander, 1846	6	17	myrmsc
Pheidole avia Forel, 1908	5	2	pheiav	*Stenamma debile* (Foerster, 1850)	-	16	stende
Pheidole lucretii Santschi, 1923	13	8	pheilu	*Temnothorax affinis* (Mayr, 1855)	-	2	temnaf
Pheidole nesiota Wilson, 2003	19	11	pheine	*Temnothorax nylanderi* (Foerster, 1850)	53	52	temnny
Pheidole risii Forel, 1892	4	3	pheiri	*Tetramorium caespitum* (Linnaeus, 1758)	-	1	tetrca
Pheidole sarcina Forel, 1912	11	9	pheisa				

IB = incidence in baits (i.e. number of sample points where the species was recorded with this method); IP = incidence in pitfall traps; - = species was not recorded with this method (double “-“ means that the species was only recorded by colony sampling).
