# Supplementary material for: Unveiling community patterns and trophic niches of tropical and temperate ants using an integrative framework of field data, stable isotopes and fatty acids
Source: PeerJ. 2018 Aug 22;6:e5467. doi: 10.7717/peerj.5467 (PMC6109374; doi:10.7717/peerj.5467)
Supplement: Supplemental Information 4 — NLFAs are ordered by decreasing contribution towards dissimilarity between sites (Bray-Curtis). Asterisks indicate statistically significant differences. [file peerj-06-5467-s004.pdf]

SIMPER analysis for ant fatty acids in Brazil and Germany.

NLFAs are ordered by decreasing contribution towards dissimilarity between sites (Bray-Curtis). Asterisks indicate statistically significant differences.

NLFA	Average dissimilarity	SD	Average/SD ratio	Average % Brazil	Average % Germany	Cumulative contribution	p
C18:1n9	0.210	0.087	2.409	30.311	72.257	0.473	0.000*
C18:0	0.091	0.040	2.312	23.231	5.039	0.679	0.000*
C16:0	0.083	0.053	1.558	33.075	17.810	0.866	0.056
C18:2n6	0.026	0.022	1.185	5.919	0.808	0.924	0.222
C16:1n9	0.009	0.007	1.346	1.182	2.047	0.944	0.250
C18:2unk1	0.006	0.006	1.001	1.634	0.500	0.958	0.665
C18:2unk2	0.005	0.005	0.930	1.187	0.222	0.970	0.590
C18:1n11	0.004	0.004	1.060	1.032	0.245	0.979	0.211
C14:0	0.003	0.002	1.556	0.875	0.474	0.985	0.024
C12:0	0.002	0.001	1.229	0.451	0.087	0.990	0.154
C20:0	0.001	0.001	1.045	0.310	0.099	0.992	0.746
C17:0	0.001	0.001	1.316	0.373	0.256	0.995	0.325
C16:1n7	0.001	0.002	0.393	0.194	0.091	0.997	0.624
C15:0	0.001	0.000	1.454	0.158	0.016	0.999	0.040*
C22:0	0.000	0.000	1.384	0.000	0.039	0.999	0.000*
iC15:0	0.000	0.000	0.453	0.033	0.000	0.999	1.000
iC17:0	0.000	0.000	0.360	0.016	0.000	1.000	1.000
C24:0	0.000	0.000	1.182	0.000	0.011	1.000	0.000*
aiC17:0	0.000	0.000	0.430	0.010	0.000	1.000	1.000
aiC15:0	0.000	0.000	0.488	0.010	0.000	1.000	1.000
