# Supplementary material for: Unveiling community patterns and trophic niches of tropical and temperate ants using an integrative framework of field data, stable isotopes and fatty acids
Source: PeerJ. 2018 Aug 22;6:e5467. doi: 10.7717/peerj.5467 (PMC6109374; doi:10.7717/peerj.5467)
Supplement: Supplemental Information 5 [file peerj-06-5467-s005.pdf]

Principal component analysis of ant species × baits in Brazil and Germany.

	PC1	PC2	PC3	PC4	PC5	PC6
**Brazil**						
Inertia/variance = 4.435						
Eigenvalue	1.61	1.05	0.85	0.46	0.29	0.17
% of variance explained	36.4	23.8	19.2	10.4	6.5	4
**Germany**						
Inertia/variance = 3.238						
Eigenvalue	1.676	0.804	0.519	0.151	0.084	0.004
% of variance explained	51.8	24.8	16	4.7	2.6	0.1

Regression values of NLFAs with the two first Principal Components in Brazil and Germany.

Asterisks indicate statistically significant differences.

NLFA	PC 1	PC 2	r^2	p	NLFA	PC 1	PC 2	r^2	p
**Brazil**					**Germany**				
C12:0	-0.96	0.29	0.11	0.33	C12:0	-0.73	-0.68	0.01	0.99
C14:0	-0.98	-0.18	0.39	0.01*	C14:0	0.35	-0.94	0.20	0.68
iC15:0	0.30	-0.96	0.12	0.29	C15:0	-0.94	-0.33	0.20	0.65
aiC15:0	0.99	-0.13	0.12	0.29	C16:1n7	-0.96	-0.27	0.52	0.18
C15:0	-0.07	-1.00	0.11	0.34	C16:1n9	-0.27	-0.96	0.49	0.28
C16:1n7	0.18	-0.98	0.19	0.15	C16:0	0.18	-0.98	0.18	0.74
C16:1n9	-0.73	-0.68	0.14	0.23	C17:0	0.74	-0.67	0.84	0.04*
C16:0	-0.41	0.91	0.26	0.06	C18:2n6	0.88	-0.48	0.08	0.79
iC17:0	0.45	-0.89	0.13	0.26	C18:1n9	-0.56	0.83	0.33	0.46
aiC17:0	0.90	-0.45	0.16	0.23	C18:1n11	0.89	-0.46	0.42	0.35
C17:0	0.79	-0.62	0.09	0.42	C18:0	1.00	0.07	0.78	0.05*
C18:2n6	0.68	0.73	0.13	0.29	C18:2unk1	-0.31	-0.95	0.08	0.84
C18:1n9	0.25	-0.97	0.32	0.02*	C18:2unk2	0.99	-0.15	0.08	0.85
C18:1n11	-0.15	-0.99	0.09	0.39	C20:0	0.99	-0.12	0.23	0.60
C18:0	-0.23	0.97	0.23	0.09	C22:0	0.91	0.42	0.33	0.43
C18:2unk1	0.98	0.21	0.31	0.03*	C24:0	1.00	0.04	0.60	0.19
C18:2unk2	0.97	0.25	0.22	0.09					
C20:0	0.58	-0.81	0.14	0.23					
