# Supplementary material for: Unveiling community patterns and trophic niches of tropical and temperate ants using an integrative framework of field data, stable isotopes and fatty acids
Source: PeerJ. 2018 Aug 22;6:e5467. doi: 10.7717/peerj.5467 (PMC6109374; doi:10.7717/peerj.5467)
Supplement: Supplemental Information 2 — Incidence means the number of sample points where a species was recorded using each method. The dashed lines indicate similar expected incidences, taking into account the total number of incidences for each method (mBR = 1.4, mGE = 0.8). [file peerj-06-5467-s002.pdf]

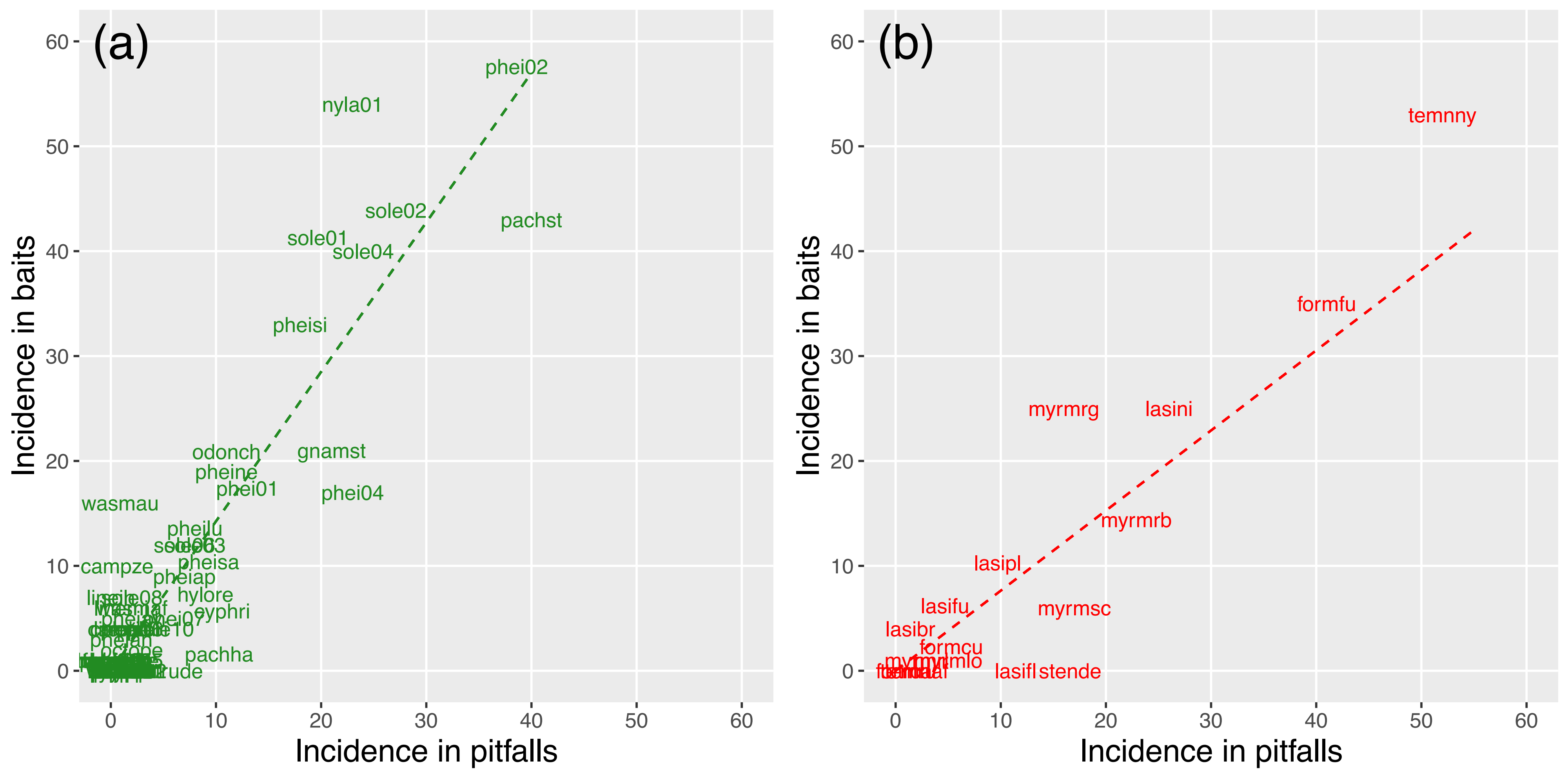

(a)
(b)
Incidence in baits
Incidence in pitfalls
0
10
20
30
40
50
60
phei02
nyla01
sole02
pachst
sole01
sole04
pheisi
odonch
gnamst
pheine
phei01
phei04
wasmau
pheilu
campze
pheisa
pheiap
hylore
eyphri
pachha
temnny
formfu
myrmrg
lasini
myrmrb
lasipl
lasifu
myrmsc
lasibr
formcu
lasifl
stende
